# Macrophage Polarization Modulated by Porcine Circovirus Type 2 Facilitates Bacterial Coinfection

**DOI:** 10.3389/fimmu.2021.688294

**Published:** 2021-07-28

**Authors:** Wen Zhang, Zhendong Fu, Hongyan Yin, Qingbing Han, Wenhui Fan, Fangkun Wang, Yingli Shang

**Affiliations:** ^1^Department of Preventive Veterinary Medicine, College of Veterinary Medicine, Shandong Agricultural University, Taian, China; ^2^Shandong Provincial Key Laboratory of Animal Biotechnology and Disease Control and Prevention, Shandong Agricultural University, Taian, China; ^3^CAS Key Laboratory of Pathogenic Microbiology and Immunology, Institute of Microbiology, Chinese Academy of Sciences, Beijing, China; ^4^Institute of Immunology, Shandong Agricultural University, Taian, China

**Keywords:** PCV2, Cap, JMJD3, macrophage polarization, bacterial coinfection

## Abstract

Polarization of macrophages to different functional states is important for mounting responses against pathogen infections. Macrophages are the major target cells of porcine circovirus type 2 (PCV2), which is the primary causative agent of porcine circovirus–associated disease (PCVAD) leading to immense economic losses in the global swine industry. Clinically, PCV2 is often found to increase risk of other pathogenic infections yet the underlying mechanisms remain to be elusive. Here we found that PCV2 infection skewed macrophages toward a M1 status through reprogramming expression of a subset of M1-associated genes and M2-associated genes. Mechanistically, induction of M1-associated genes by PCV2 infection is dependent on activation of nuclear factor kappa B (NF-κB) and c-jun N-terminal kinase (JNK) signaling pathways whereas suppression of M2-associated genes by PCV2 is *via* inhibiting expression of jumonji domain containing-3 (JMJD3), a histone 3 Lys27 (H3K27) demethylase that regulates M2 activation of macrophages. Finally, we identified that PCV2 capsid protein (Cap) directly inhibits *JMJD3* transcription to restrain expression of interferon regulatory factor (*IRF4*) that controls M2 macrophage polarization. Consequently, sustained infection of PCV2 facilitates bacterial infection *in vitro*. In summary, these findings showed that PCV2 infection functionally modulated M1 macrophage polarization *via* targeting canonical signals and epigenetic histone modification, which contributes to bacterial coinfection and virial pathogenesis.

## Introduction

Host organisms are generally exposed to multiple pathogens simultaneously in a natural environment (1). Hence, polymicrobial infection or coinfection are common in the infectious diseases that can lead to severity and complications in clinical treatment. Many well-recognized instances involving coinfection of viruses and secondary bacteria are known in both human and animals (2, 3). For example, influenza virus and bacterial pneumonia combined are among the most deadly infection in human cases (1, 4–6). Swine viruses such as porcine reproductive and respiratory syndrome virus (PRRSV) or PCV2 infection often predisposes pigs to bacterial coinfection (7–12). However, how the host immune responses to one pathogen alters the immune response to another infectious agent in host-pathogen interactions remain largely unclear.

PCV2 is the primary causative agent of PCV-associated diseases, leading to immense economical losses in the swine industry worldwide (13–15). Notably, concurrent infections with other swine pathogens are usually seen in most field PCVAD cases, which exacerbates the pathogenesis of diseases (13, 16–18). The most frequently observed bacteria in PCVAD cases are *Actinobacillus pleuropneumoniae* (APP), *Glaesserella parasuis* and *Streptococcus suis* (9, 10, 15, 19, 20). As the smallest virus known to infect mammal, PCV2 has a single-strand circular DNA genome approximately 1.7 kb in size and preferentially targets the swine immune systems. Therefore, PCV2 replication heavily depends on the immune cell machinery and interacts with many host cellular factors. In addition, PCV2 can persist in pig and resides in certain immune cells, such as macrophages and dendritic cells, and modulates their functions (14). It is believed that PCV2 modulation of the innate immune responses may render the pigs more susceptible to secondary or concurrent viral or bacterial infections. However, the underlying mechanisms of increased susceptibility by PCV2 infection to subsequent bacterial infection are poorly understood.

Macrophages are innate immune cells that have a central role in detecting pathogen infections. Infection or tissue injury typically activate macrophages to produce inflammatory cytokines or chemokines to initiate host defense. Functionally, activation of macrophages polarizes towards two phenotypes: classical activation (also termed M1) and alternative activation (M2) (21, 22). While M1 macrophages are effective at host defense, M2 macrophages are important for resolution of inflammation and tissue repair (21). Due to the critical roles of macrophage functions in host defense and inflammation resolution, macrophage polarization is dynamic regulated by multiple mechanisms including cytokines, signaling pathways, transcription factors as well as epigenetic modulation (23–26). Several studies have demonstrated that macrophage polarization is associated with viral infections, such as influenza virus and human immunodeficiency virus, and polarized macrophages contribute to the pathogenesis of diseases (27–30). However, little is known about the acquisition and maintenance mechanisms of macrophage programming.

In this study, we explored the role of PCV2 infection in modulation of macrophage polarization and functions in primary macrophages and in viral infection conditions *in vivo*. We found that PCV2 infection shifted macrophage towards a M1 status *via* reprogramming gene transcriptome of macrophages. Moreover, PCV2 infection restrained expression of key transcription factors for M2 polarization, which contributes to M1 phenotype maintenance. Consequently, M1 polarized macrophages promoted bacterial coinfection. Thus, our findings reveal a novel mechanism of PCV2 mediated-immunosuppression and deepen our understanding of pathogen coinfection in PCVAD cases and provide potential disease-control strategies.

## Materials and Methods

### Mice

C57BL/6 mice were purchased from Beijing Vital River Laboratory Animal Technology Co., Ltd and mice with 6-8 weeks of age were used for all experiments. *MyD88^-/-^* mice were kindly provided by Dr. Hua Tang (Shandong First Medical University, China). All animal protocols were reviewed and approved by Shandong Agricultural University Animal Care and Use Committee (Approval Number: # SDAUA-2018-057) and were performed according to the Animal Ethics Procedures and Guidelines of the People’s Republic of China (31).

### Cells and Cell Culture

Murine bone marrow-derived macrophages (BMDMs) were obtained as previously described (32) and maintained in Dulbecco’s modified eagle medium (DMEM) supplemented with 10% fetal bovine serum (FBS, Gibco, USA) and 10% L929 cell supernatant as conditioned medium providing macrophage colony stimulating factor. Immortalized BMDMs (iBMDM), Plat-E and HEK293T were cultured in DMEM supplemented with 10% FBS (Biological Industries, Israel). Porcine alveolar macrophages (PAMs) obtained by bronchoalveolar lavages from the lungs of two specific pathogen free piglets as previously described (33). Briefly, the lung was removed immediately after the pigs were euthanized and washed three times with cold, sterile calcium and magnesium-free phosphate-buffered saline (PBS) supplemented with 0.2% ethylene diamine tetraacetic acid (EDTA). The wash media were collected and poured through a layer of gauze into a sterile bottle to remove mucus. The collected liquid was then centrifuged at 300 × g at 4°C for 10 min and cultured in RPMI 1640 medium (Gibco, USA) with 10% FBS (Gibco, USA), 100 U/ml penicillin and 100 μg/ml streptomycin.

### Virus Infection and Bacterial Coinfection

The PCV2 strain (IDSDTA2017-1, GenBank: MN400446.1) was isolated from pig sources in Shandong, China and was identified as PCV2b subtype by sequencing (34). A virulent APP (Serotype 1) strain was purchased from China Veterinary Culture Collection Center (Cat# CVCC259, Beijing, China) and was cultured on tryptic soy agar (TSA) or in tryptic soy broth (TSB) supplemented with 10 μg/mL nicotinamide adenine dinucleotide (NAD) and 10% FBS at 37°C. *Salmonella* Tyhphimurium (STM) strain (CDC 6516-60) was purchased from American Tissue Culture Collection (Cat# 14028) and was cultured on Luria Bertani (LB) at 37°C. APP and STM strains were transformed with a bacterial green fluorescent protein (GFP) expression vector (pTurboGFP-B, Evrogen, Russia) to generate APP-GFP and STM-GFP strains by using electroporation method as described previously (35). For *in vitro* viral infection, BMDMs were infected with PCV2 (MOI=0.02) for various times at 3, 6, 12 or 24 h or were infected with different doses of PCV2 (MOI=0.02, 0.05 and 0.1) for 12 h, or PAMs were infected with PCV2 (MOI=0.02) for 24 h before further analysis for mRNA or protein expression. For *in vitro* coinfection, cells were infected with PCV2 (MOI=0.02) for 12 h and then inoculated with APP, STM, APP-GFP or STM-GFP (MOI=10) for another 3 h. For *in vivo* infection, mice were mock-infected or intraperitoneally infected with PCV2 (5×10^5^ pfu/mouse) for 12 h to examine gene expression in peritoneal cells (n=4 in each condition) or for 24 h to analyze population of polarized macrophages in peritoneal cavity (n=6 in each condition).

### Inhibition of Signaling Pathways

For blocking of MyD88-dependent activation of NF-κB and MAPK signals, BMDMs from wild type and *MyD88* ^-/-^ mice were infected with PCV2 (MOI=0.02) for different times to examine the effect of NF-κB and MAPK activation in gene regulation of macrophages. Two mice were used in each group. Specific chemical inhibitors were also used to inhibit activation of NF-κB and MAPKs. Briefly, BMDMs from wild type mice were pretreated with NF-κB inhibitor BAY11-7082 (BAY11, 10μM), JNK inhibitor SP600125 (SP, 10μM), MEK inhibitor U0126 (10μM) or p38 MAPK inhibitor SB203580 (SB, 10μM) for 1 h prior to virus infection (36). All chemical inhibitors were purchased from MedChemExpress (MCE, New Jersey, USA).

### Reverse Transcription and Quantitative Real-Time PCR (qPCR)

RNA was extracted from whole-cell lysates with a total RNA purification kit (GeneMarkbio) and reversely transcribed to cDNA with Reverse Transcriptase M-MLV (Takara). qPCR was performed using UltraSYBR Mixture (Cwbio, China). Threshold cycle numbers were normalized to triplicate samples amplified with primers specific for *GAPDH* or *β-ACTIN*. qPCR primer sequences are listed in the **Supplementary Table 1**.

### Isolation of Peritoneal Cells

Peritoneal cells were isolated as previously described with minor modification (37). Briefly, peritoneal cells were washed out with ice-cold PBS containing 2 mM EDTA. After washing twice with PBS, cells were used for total RNA extraction or further analysis.

### RNA Sequencing (RNA-Seq) Analysis

For RNA-seq analysis, BMDMs were left untreated or infected with PCV2 (MOI=0.02) for 12 h. Cells were then harvested for total RNA extraction with a total RNA purification kit (GeneMarkbio). RNA quality was analyzed by Agilent 2100 Bioanalyzer (Agilent Technologies, CA, USA). 1 μg of total RNA with RNA integrity number (RIN) value above 9 in this study was converted into RNA-seq quantification libraries. RNA-seq libraries with different indices were multiplexed and loaded on an Illumina HiSeq X Ten at GENEWIZ China per the manufacturer’s recommended protocol. Pair end RNA-seq reads were aligned to mouse genome mm10 *via* software Hisat2 (v2.0.1) and only uniquely mapped reads were preserved. For coverage of mapped RNA-seq reads in transcripts, the expression level of each genes transcripts was calculated as the normalized fragment count to fragments (FPKM). Differential expression analysis of genes between experimental conditions was implemented using the Bioconductor software package edgeR (V3.4.6) (38). The p-values were false discovery rate (FDR) corrected by using the Benjamini-Hochberg approach (39). Genes with adjust P value <0.05 and fold change ≥2 between two conditions were regarded as significantly up-regulated genes, and significantly down-regulated genes were identified with P value <0.05 and fold changes ≤0.5 between two conditions. Two replicates in each condition were performed. The RNA-seq data reported in this study have been deposited in the Gene Expression Omnibus with the accession number GSE171721.

### Cloning and Expression of PCV2-Encoded Proteins

PCV2 ORF1 or ORF2 were amplified by PrimeSTAR HS DNA Polymerase (Takara Bio, Beijing, China) with PCV2 DNA as a template, and cloned into the pCMV-myc expression vector. The ORF1 and ORF2 of PCV2 with N-terminal myc tag were amplified by PrimeSTAR HS DNA Polymerase with pCMV-ORF1 or pCMV-ORF2 vectors as a template, followed by BamH I and Xho I (ORF1) or BamHI and Not I (ORF2) digestion and were ligated with pMx-puro retroviral vectors. The primers used to construct these ORFs are listed in the **Supplementary Table 1**. All clones were sequenced and expression was confirmed by immunoblotting transiently transfected HEK293T cell lysates with an anti-myc antibody (Santa Cruz Biotechnology, USA).

### Flow Cytometry

Peritoneal cells were stained with a PE/Cy7-conjugated anti-mouse F4/80 (1:200, Cat#25-4801-82, eBioscience) and APC-eFluor780-conjugated anti-mouse CD11b (1:200, Cat#47-0112-82, eBioscience) for gating macrophages. PE/Cy7-conjugated anti-mouse F4/80 together with eFluor450 anti-mouse Ly6C (1:800, Cat#48-5932-80, eBioscience) was used to stain M1 phenotype macrophages and PE/Cy7-conjugated anti-mouse F4/80 together with FITC-conjugated anti-mouse CD206 (1:200, Cat#141704, Biolegend) was used to stain M2 phenotype macrophages as previously described (30). Briefly, cells were first blocked with anti-mouse CD16/CD32 (1:200, Cat#14-0161-81, eBioscience) to eliminate of Fc receptor-mediated antibody binding. Cell surface staining was performed by incubating cells with indicated antibodies for 15 min on ice. CD206 staining was carried out using the intracellular staining kit (Cat#00-5523-00, eBioscience) according to the manufacturer’s protocol. Cells were washed and analyzed on an LSR Fortessa flow cytometer (BD Biosciences, USA) using FlowJo software (BD Biosciences, USA).

### Bacterial Infection Assay and Fluorescence Microscope

For APP or STM coinfection, cells were washed with PBS to remove unattached bacteria and then lysed in 0.1%Triton X-100 for 5 min, the lysate was serially diluted in PBS and plated onto TSB or LB-Agar for overnight before bacterial colonies was counted and colony formation unit (CFU) in each plate was calculated. GFP positive cells were analyzed by flow cytometry after washing with PBS. For immunofluorescence assay, cells were fixed with 4% paraformaldehyde for 20 min at room temperature after removal of unattached bacteria and were stained with a red fluorescence probe Dil (C1036, Beyotime, China) and DAPI (C1005, Beyotime, China) to label cell membrane and nucleus, respectively. Images were acquired on a Nikon Ni-U fluorescence microscope using Nis-elements software (Nikon, Japan).

### Immunoblotting Analysis

Whole-cells lysates were prepared as described previously (37). For immunoblotting analysis, lysates were separated by 12% SDS-­PAGE and transferred to a PVDF membrane (Millipore) for probing with specific antibodies. Antibody against IκBα (1:1,000, 4814), p-p38(1:1,000, 9215), ERK (1:1,000,9102), p-ERK (1:1,000, 9101), JNK (1:1,000, 9252) and p-JNK (1:2,000, 9255) were obtained from Cell Signaling Technology. Antibody against p38 (1:1,000, sc-7972) and myc (1:1,000, sc-40) were purchased from Santa Cruz Biotechnology and JMJD3 (1:500, NBP1-06640SS) was purchased from Novus Biologicals. Anti-PCV2 Cap polyclonal antibody was previously generated (34).

### Chromatin Immunoprecipitation (ChIP) Assay

ChIP assay was performed as described previously (32). Briefly, BMDMs were left untreated or infected with PCV2 (MOI=0.02) for 12 h. BMDMs were fixed with 1% methanol-free formaldehyde (Thermo Scientific) for 5 min at 22~25°C followed by quenching with 125 mM glycine for another 5 min. Cells were then lysed in 1% SDS lysis buffer. Chromatin DNAs were sheared to an average of 300 bp by using a bioruptor plus (Diagenode, Belgium). For immunoprecipitations, the following antibodies were used: anti-H3K27me3 (5 μg/sample, ab6002, Abcam), anti-pol II (2.5 μg/sample, sc-47701x, Santa Cruz) and anti-myc (2 μg/sample, sc-40x, Santa Cruz). After purification, immunoprecipitated DNAs were analyzed by qPCR with corresponding primers (sequences are listed in the **Supplementary Table 1**) and relative occupancies were normalized to input DNA. Three independent experiments were performed in each ChIP assay and cumulative data were pooled.

### Dual-Luciferase Reporter Assay

Murine *JMJD3* reporter plasmid was constructed by subcloning the *JMJD3* promoter sequences (from positions -1000 to 0) into a luciferase expression vector pGL3-basic. HEK293T were cotransfected in duplicates with the *JMJD3* reporter plasmid and an expression plasmid encoding PCV2 Rep (pCMV-myc-ORF1) or Cap proteins (pCMV-myc-ORF2) or a control vector (pCMV-myc) using Lipofectamine 2000 reagent (Invitrogen). Twenty-four hours after transfection, cells were harvested and cell lysates were prepared and analyzed using Dual-Luciferase Report Assay System (Promega). The renilla luciferase reporter gene (pRL-TK, Promega) was used as an internal control.

### Retroviral Transduction

Retroviral transduction was performed as previously described (40). Briefly, Plat-E cells (4 × 10^6^) were seeded into 100 cm plates and were cultured for 24 h. Cells were then transfected with 15 µg of retroviral vectors of pMx-Puro-myc-ORF1, pMx-Puro-myc-ORF2 or pMx-control by using Lipofectamine 2000 reagent (Invitrogen) for 48 h. Viral supernatants were collected and filtered, and 5 ml of viral supernatant was used to transduce 5 × 10^6^ BMDMs in presence of 6 µg/ml of polybrene (Solarbio, China) for 24 h. BMDMs were selected by 2 µg/ml puromycin (Solarbio, China) for 3 d and then were used for experiments.

### Statistical Analysis

*P* values were calculated with a two-tailed paired or unpaired Student’s *t* test by Prism GraphPad software (v5.0). *P* values of ≤0.05 were considered significant.

## Results

### PCV2 Infection Shifts Macrophage Polarization Status *In Vitro*

**Figure 1:**
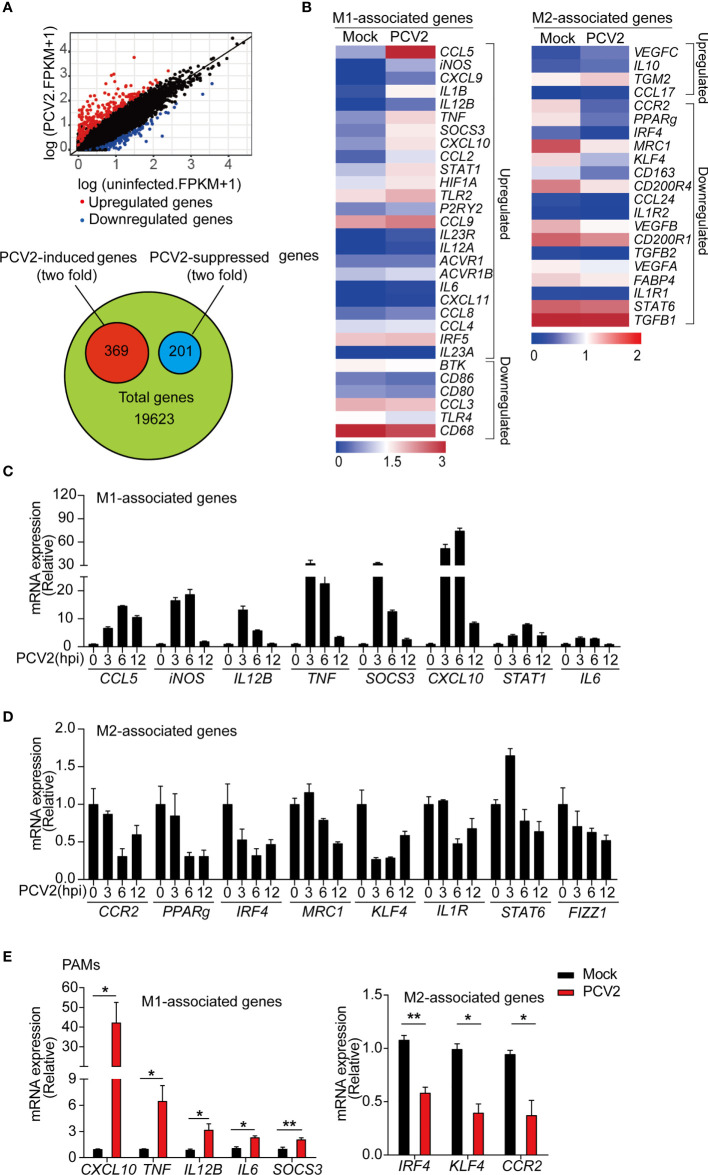
PCV2 infection induces M1 macrophage polarization *in vitro*. **(A)** Scatter blot of RNA-seq analysis of differentially expressed genes in BMDMs left untreated or infected with PCV2 (MOI=0.02, the same dose below) for 12 h (top) and quantification of PCV2-induced genes or PCV2-suppressed genes in BMDMs (bottom). **(B)** Heatmap of M1-associated genes (left) and M2-associated genes (right) regulated by PCV2 infection in BMDMs. **(C, D)** Quantitative real-time PCR (qPCR) analysis of mRNA expression of representative M1-associated genes **(C)** or M2-associated genes **(D)** in BMDMs infected with PCV2 at indicated periods. **(E)** qPCR analysis of mRNA expression of M1-associated genes (left) and M2-associated genes (right) in PAMs infected with PCV2 for 24 h. Data are representative of one experiment **(A, B)** or three (**C, D**; mean + s.d. of technical triplicates) independent experiments or pooled from three (**E**, mean + s.d.) independent experiments. *P < 0.05; **P < 0.01 (Student’s *t* test).

Macrophages play critical roles in host defense against infection. In response to various stimuli or diverse signals, macrophages can alter their phenotype on activation to two functional polarization states: the pro-inflammatory M1 phenotype and the anti-inflammatory M2 phenotype (26). Upon viral infection, macrophages generally exhibit the M1 phenotype to release inflammatory mediators. However, excessive or prolonged M1 phase can cause tissue damage and contribute to pathogenesis (41, 42). To know the influences of PCV2 infection on macrophage phenotype, we first examined the expression profiling of global genes in PCV2 infected-BMDMs by RNA-seq. The results revealed that expression of 369 genes were greater than twofold in PCV2 infected-BMDMs relative to their expression levels in control cells (**Figure 1A**). In addition, the expression of 201 genes were lower than in PCV2-infected macrophages compared to that of control cells (**Figure 1A**). Among these genes, we found that PCV2 infection strikingly induced expression of a subset of M1-associated genes including *CXCL10*, *SOCS3*, *iNOS*, *IL12B* and *STAT1* whereas decreased expression of multiple M2-associated genes such as *IRF4*, *KLF4*, *PPARg* and *CCR2* (**Figure 1B**), indicating that PCV2 infection likely reprograms the macrophage transcription phenotype. To further determine the result obtained by RNA-seq analysis, we tested the expression of M1 and M2 macrophage-associated genes in BMDMs infected with PCV2 for various times or with different doses of PCV2. qPCR analysis showed that PCV2 infection promoted the expression of key M1 macrophage-associated genes and suppressed the expression of M2 macrophage phenotype genes (**Figures 1C, D** and **Supplementary Figures 1A–C** in **Supplementary Material**), which indicated that PCV2 infection induced M1 macrophages polarization state in BMDMs. Consistent with the results observed in BMDMs, PCV2 infection markedly increases M1-associated genes and inhibits M2-associated genes in PAMs (**Figure 1E**), suggesting that PCV2 infection also induces M1 macrophage polarization state in PAMs and such phenomenon is not restricted in murine cells. Together, these results demonstrate that PCV2 infection induces M1 macrophage polarization state *in vitro*.

### PCV2 Infection Induces M1 Macrophage Polarization *In Vivo*

**Figure 2:**
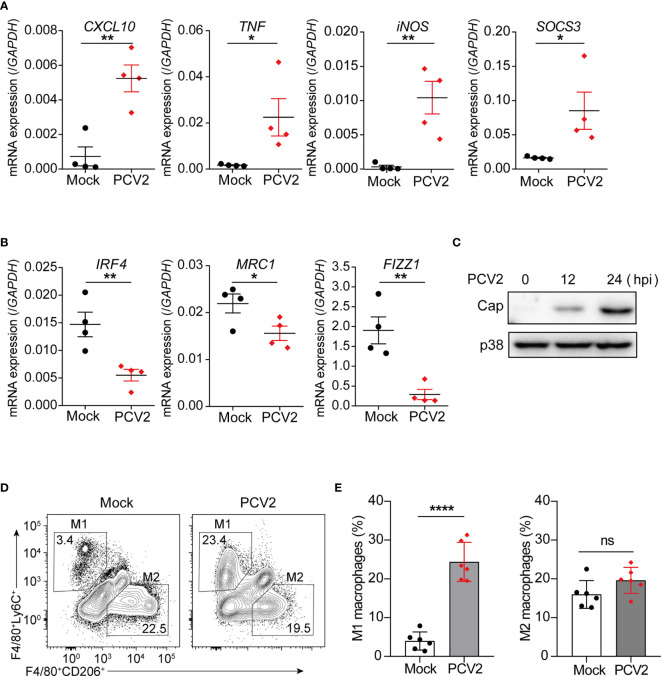
PCV2 infection promotes M1 macrophage polarization *in vivo*. **(A, B)** qPCR analysis of mRNA expression of M1-associated genes **(A)** and M2-associated genes **(B)** in peritoneal cells from mice (n=4) were either mock-infected or infected with PCV2 (5×10^5^ pfu per mouse, the same dose below) for 12 h. **(C)** Immunoblot analysis expression of PCV2 Cap in peritoneal cells from mice infected with PCV2 for indicated periods. **(D, E)** Flow cytometry analysis of peritoneal exudates from mock mice or PCV2-infected mice for 24 h. Numbers in top left gated population indicate percent F4/80^+^Ly6C^hi^ cells (M1-polarized macrophages) and numbers in lower right gated population indicate percent F4/80^+^CD206^hi^ cells (M2-polarized macrophages). Right, cumulative quantification of M1 and M2 macrophages as at left panels. Each symbol represents an individual mouse. Data are from one experiment (**A–E**; mean + s.d of n=4 mice in **A** and **B** or n=6 mice in **E**) *P < 0.05; **P < 0.01; ****P < 0.0001 (Student’s *t* test). ns, not significant.

Having known that PCV2 infection promotes macrophage polarization towards M1 phenotype *in vitro* in cultured BMDMs and PAMs, we next investigated whether such phenomenon happened *in vivo*. qPCR analysis showed that expression levels of the M1-associated genes, such as *CXCL10*, *TNF*, *iNOS*, *SOCS3*, were significantly increased in peritoneal cells from PCV2-infected mice compared with that of control mice (**Figure 2A**), implying that PCV2 infection likely induces M1 phenotype of macrophages *in vivo.* Meanwhile, PCV2 infection suppressed expression of key M2-associated genes such as *IRF4*, *MRC1* and *FIZZ1* in peritoneal cells (**Figure 2B**). In addition, successful infection of PCV2 *in vivo* was further confirmed as expression of Cap protein was examined in peritoneal cells from PCV2-infected mice (**Figure 2C**). A previous study suggested that the F4/80^+^Ly6c^+^ double positive cells are M1 macrophages and F4/80^+^CD206^+^ double positive cells are M2 macrophages in murine peritoneal cells (30). Therefore, we further examined the M1 and M2 macrophage population in peritoneal cells from control mice or PCV2-infected mice. Flow cytometry analysis showed that number of M1 macrophages dramatically increased in peritoneal cavity from PCV2-infected mice (**Figures 2D, E**, left panels), suggesting that PCV2 infection indeed increases M1 macrophages *in vivo*. In contrast, PCV2 infection did not alter the number of M2 macrophages *in vivo* (**Figures 2D, E**, right panels). Moreover, induction of M1-associated genes was also observed in liver and spleen from PCV2 infected-mice (**Supplementary Figures 2A, B** in **Supplementary Material**). Taken together, these data suggest that PCV2 infection promotes M1-associated gene expression, leading to increase of M1-ploarized macrophages *in vivo*.

### Induction of M1-Associated Genes by PCV2 Is Through the Activation of NF-κB and JNK

**Figure 3:**
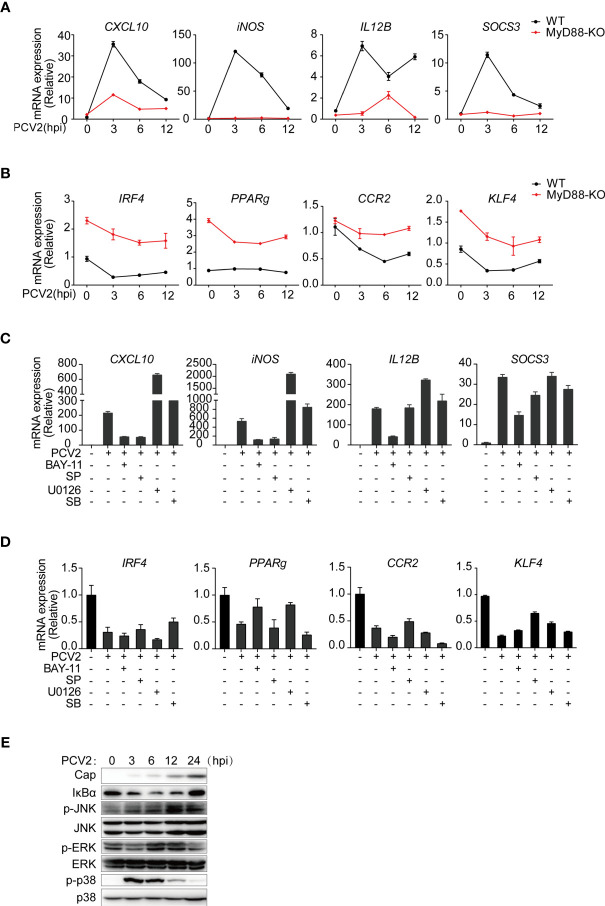
PCV2 regulates M1-polarized macrophages *via* activation of NF-κB and JNK signals. **(A, B)** qPCR analysis of mRNA expression of key M1-associated genes **(A)** or M2-associated genes **(B)** in BMDMs obtained from wild-type and *MyD88*-deficient infected with PCV2 (MOI=0.02, the same dose below) for indicated periods (horizontal axes). **(C, D)** qPCR analysis of mRNA expression of key M1-associated genes **(C)** or M2-associated genes **(D)** in BMDMs. Cells were left untreated or infected with PCV2 alone for 6 h, or pretreated with NF-κB inhibitor (BAY11-7082,10μM), JNK inhibitor (SP600125,10μM), ERK inhibitor (U0126,10μM) or p38 inhibitor (SB203580, 10μM) for 1 h followed by PCV2 infection for 6 h. **(E)** Immunoblot analysis of total NF-κB inhibitor IκBα and phosphorylated (p-) and total ERK, JNK and p38, in whole-cell lysates of BMDMs infected with PCV2 for various times (above lanes). Data are representative of two (**A–E**; mean + SD of technical triplicates in **A–D**) independent experiments.

Next, we sought to investigate the mechanisms by which PCV2 induced expression of M1-associated genes. It has been well established that activation of NF-κB and mitogen-activated protein kinases (MAPKs) contribute to inflammatory gene activation. Moreover, NF-κB and MAPK signals have been reported to be activated in PCV2 infected-PAMs or PK-15 cells (33, 43, 44). Given that most of the M1-associated genes related to PCV2 infection are pro-inflammatory mediators, we therefore examined whether PCV2-induced expression of M1-associated genes was dependent on myeloid differentiation factor 88 (MyD88), the key adaptor protein that mediates downstream activation of NF-κB and MAPKs. Upon PCV2 infection, induction of M1-associated genes was significantly suppressed without affecting expression of M2-associated genes in *MyD88*-deficicent macrophages (**Figures 3A, B** **)**, suggesting that PCV2-induced expression M1-associated genes is through MyD88-mediated signaling pathways. In line with this notion, inhibition of NF-κB or JNK by chemical inhibitors dramatically suppressed expression of M1-associated genes without affecting expression of M2-associated genes (**Figures 3C, D** **)**, demonstrating that PCV2 infection alter expression of M1-associated genes but not M2-associated genes *via* canonical NF-κB and MAPK signals. Consistently, PCV2 infection induced activation of NF-κB and MAPKs in macrophages (**Figure 3E**). Together, these results demonstrated that activation of NF-κB and JNK is critical of PCV2-mediated induction of M1-associated genes in macrophages.

### PCV2 Inhibits Expression of M2-Associated Genes *via* Histone Modification

**Figure 4:**
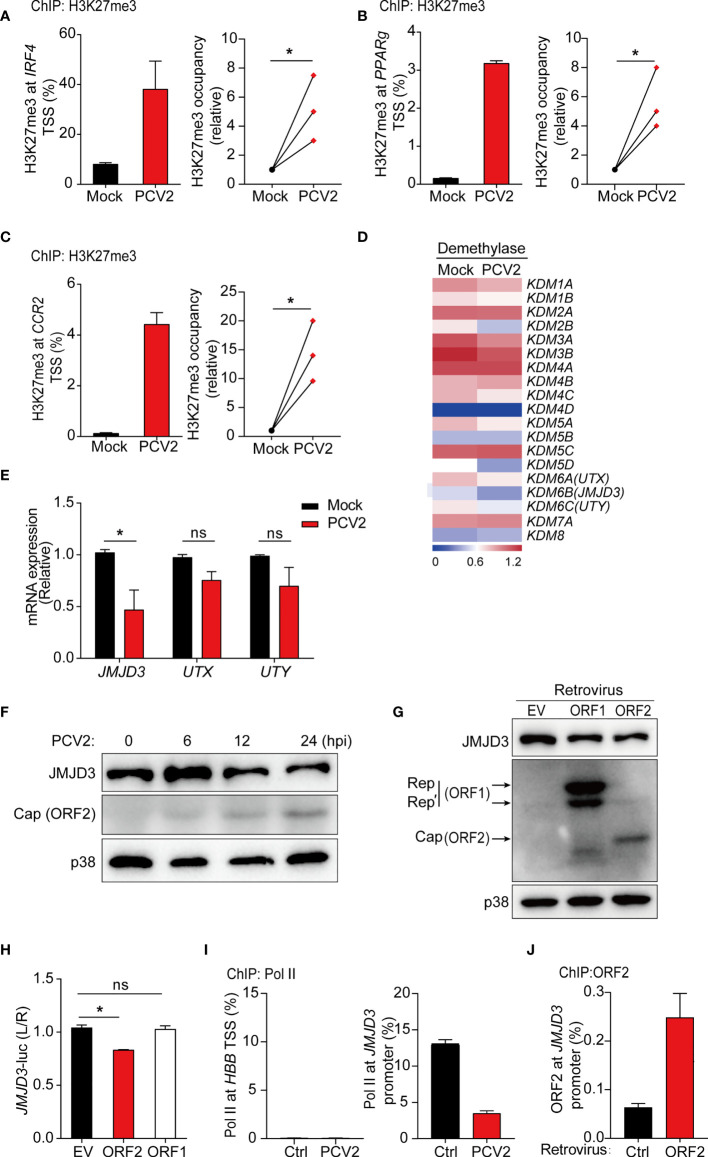
PCV2 enhances H3K27 trimethylation to suppress M2-associated gene expression. **(A–C)** ChIP analysis of H3K27me3 occupancy at the promoter regions of *IRF4* **(A)**, *PPARg* **(B)** and *CCR2* **(C)** in BMDMs left untreated or infected with PCV2 (0.02 MOI, the same dose below) for 12 h, and cumulative results (**A–C**, right) for H3K27me3 at *IRF4, PPARg* or *CCR2* under PCV2-infected conditions, presented relative to results obtained from control cells, set as 1. **(D, E)** Heatmap of gene expression profiles of 19 histone demethylases **(D)** or qPCR analysis of mRNA expression of KDM6 family members (*JMJD3, UTX, UTY*) **(E)** in BMDMs left untreated or infected with PCV2 for 12 h. **(F, G)** Immunoblot analysis of JMJD3, Cap and/or Rep of PCV2 in BMDMs infected with PCV2 for various times at 6, 12 or 24 hours **(F)** or transduced with control empty vector, PCV2 ORF1 (Rep) or ORF2 (Cap)-encoding retrovirus **(G)**. p38 served as a loading control. **(H)** Luciferase activities in HEK293T cells co-transfected with a *JMJD3* promoter-driven reporter construct and ORF1 or ORF2 expression plasmid or control empty vector. Twenty-four hours after transfection, cell lysates were analyzed for luciferase activity. **(I, J)** ChIP analysis of Pol II occupancy **(I)** or PCV2 Cap (ORF2) occupancy **(J)** at the promoter of *JMJD3* (right) or the silent locus encoding hemoglobin-β (*HBB*) (left) in BMDMs left untreated or infected with PCV2 for 12 h or BMDMs transduced with control retroviruses or retroviruses expressing PCV2 ORF2 (myc tag). Data are representative from one (**D, G, J**; mean + s.d. of technical triplicates in **J**), two (**F, I**; mean + s.d. of technical triplicates in I) or three (**A–C**, mean + s.d. of technical triplicates in **A–C** left) independent experiments or pooled from three (**A–C** right, **E, H**; mean + s.d) independent experiments. ns, not significant; *P < 0.05 (Student’s *t* test).

Emerging evidence has revealed an important role for epigenetic modulation of chromatin states in regulating macrophage polarization and function (45). Histone methylation is important for M2 polarization (46). For example, histone demethylase JMJD3 facilitates expression of the key M2-promoting transcription factor *IRF4* by removing negative histone H3 lysine-27 trimethylation (H3K27me3) mark at *IRF4* locus (47). Since inhibition of NF-κB and MAPK signals does not alter PCV2-mediated suppression of M2-associated genes in macrophages, we therefore investigated whether PCV2 infection modulated histone modification on M2-associated genes. Previous study suggested that M2-macrophage marker genes were epigenetically regulated by H3K27me3, which is linked to silencing of gene expression (48). Therefore, we investigated whether PCV2 infection impaired the level of H3K27me3 on M2-associated genes by using chromatin immunoprecipitation (ChIP) assay. In resting BMDMs, occupancy by H3K27me3 modification were observed in gene promoter regions of M2-associated genes, such as *IRF4* (47), indicating that M2-associated genes are likely regulated by histone methylation. Indeed, ChIP assays revealed that occupancy of H3K27me3 modification at promoter regions of M2-associated genes including *IRF4, PPARg*, and *CCR2* was relatively higher in response to PCV2 infection in multiple independent experiments (**Figures 4A–C**), demonstrating that PCV2 infection elevated the levels of H3K27me3 modification to suppress expression of M2-associated genes.

### PCV2 Suppresses Expression of JMJD3 to Regulate H3K27 Trimethylation

To identify that how PCV2 infection modulates H3K27me3 modification at M2-associated genes, RNA-seq data were further analyzed to examine expression levels of histone demethylases and methylases in control and PCV2-infected BMDMs. While similar gene expression of histone methylases was observed in PCV2-infected BMDMs and control cells (**Supplementary Figure 3A** in **Supplementary Material**), gene expression of histone demethylase *JMJD3* in macrophages was strikingly decreased in response to PCV2 infection (**Figure 4D**). Interestingly, it has been reported that increase of JMJD3 (also known as KDM6B) contributes to transcriptional activation of M2 marker genes and the JMJD3-IRF4 axis is necessary for M2 macrophage polarization (47). Given that PCV2 infection suppresses *IRF4* expression in macrophages, we therefore sought to know whether PCV2 infection inhibit M2 macrophage polarization *via* JMJD3-IRF4 axis. In line with this hypothesis, downregulation of JMJD3 was further confirmed at both mRNA and protein level in PCV2-infected BMDMs at multiple time periods (**Figures 4E, F** **)**, suggesting that PCV2 infection suppresses JMJD3 expression. In contrast, PCV2 infection did not significantly alter the gene expression of *UTX* (also known as *KDM6A*) and *UTY* (*KDM6C*), two H3K27 demethylases belonging to the same family with JMJD3 that catalyze the conversion of H3K27me3 to H3K27me1 (**Figure 4E**), and did not affect the mRNA expression of *EZH1* and *EZH2*, two methyltransferases that catalyze H3K27me3 on gene promoters (**Supplementary Figure 3B** in **Supplementary Material**), indicating that PCV2 infection particularly inhibits expression of JMJD3 demethylase. Taken together, the above data revealed that suppression of M2-associated genes by PCV2 infection are likely through repression of JMJD3 to modulate H3K27me3.

### PCV2 ORF2, but Not ORF1, Inhibits JMJD3-IRF4 Axis

Next, we sought to investigate which components of PCV2 play critical roles in regulation of JMJD3 expression. It is well known that PCV2 ORF1 and ORF2 are two major viral encoded genes that express Rep and Cap proteins, respectively. We therefore overexpressed PCV2 ORF1 and ORF2 in BMDMs by retroviral transduction. Enforced expression of Cap, but not Rep, extensively decreased protein expression of JMJD3 (**Figure 4G**), suggesting that PCV2 Cap functions as a negative regulator of JMJD3 in macrophages. As we have observed that PCV2 infection inhibits *JMJD3* transcription, we next investigated whether Cap suppressed *JMJD3* transcription through the use of luciferase assays with a *JMJD3* reporter construct. Indeed, Cap, but not Rep, did inhibit *JMJD3*-promoter-driven luciferase activity in HEK293T cells (**Figure 4H**), which indicated that Cap protein was the key viral component for PCV2-mediated suppression of *JMJD3* transcription. Moreover, we tested whether PCV2 suppressed the *JMJD3* transcription by using ChIP assays to assess binding of Polymerase II (Pol II) to the TSS region of *JMJD3* promoter. While maximal Pol II occupancy was observed at the *JMJD3* promoter region in resting BMDMs, occupancy of Pol II markedly decreased followed by PCV2 infection (**Figure 4I**, right), further confirming that PCV2 regulates Pol II recruitment to mediate *Jmjd3* suppression. Lack of Pol II occupancy at the silent locus encoding hemoglobin-ß served as a negative control (**Figure 4I**, left). These results were consistent with the finding that Cap directly bound to the *JMJD3* promoter (**Figure 4J**). Collectively, these data suggested that PCV2-encoded Cap suppressed *JMJD3* expression *via* directly binding to the gene promoter thereby targeting H3K27me3 modification.

### PCV2 Infection Promotes Bacterial Infection *In Vitro*

**Figure 5:**
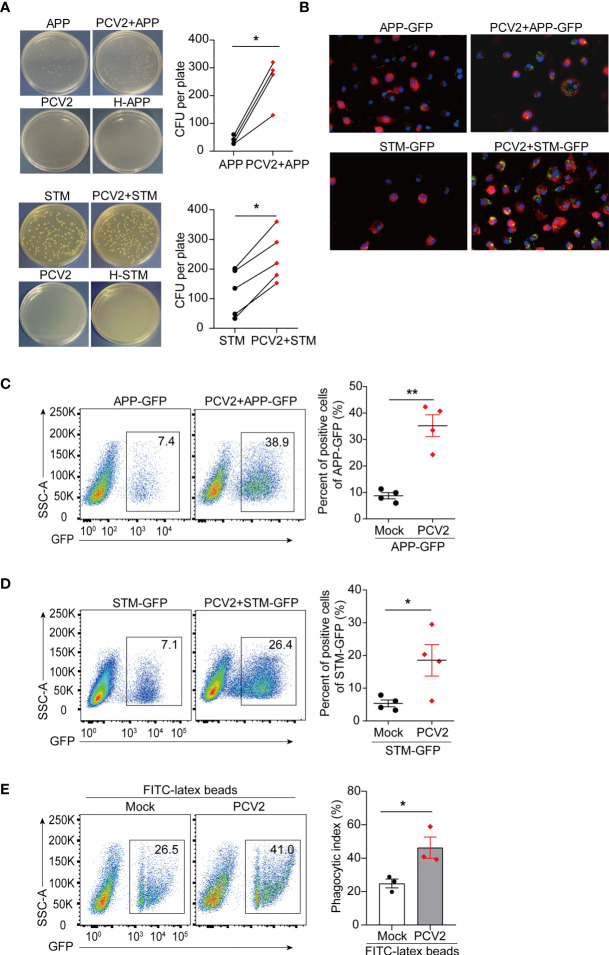
PCV2 priming facilitates bacterial infection in macrophages. **(A)** CFU of APP or STM in PCV2-infected BMDMs or control cells. Macrophages were infected with APP or STM for 3 h at a dose of MOI=10 (the same dose below). H-APP/STM: bacteria were heated at 70°C for 30 min to kill all bacteria as a negative control. Cumulative data were pooled from four or five independent experiments (right, mean + s.d.). **(B)** Fluorescence microscope of GFP positive BMDMs infected with APP-GFP or STM-GFP alone for 3 h or infected with PCV2 for 12 h followed by APP-GFP or STM-GFP for another 3 h. Cell membranes were labelled with a red fluorescence probe Dil (red) and nucleus were stained with DAPI (blue). **(C, D)** FACS analysis of GFP positive cells in BMDMs infected with APP-GFP **(C)** or STM-GFP **(D)** alone or infected with PCV2 for 12 h followed by APP-GFP or STM-GFP for another 3 h. Cumulative data are from four independent experiments (right, mean + s.d.). **(E)** FACS analysis of green fluorescence positive cells in BMDMs left untreated or infected with PCV2 for 12 h followed by inoculation with FITC-labeled latex beads for 30 min. Data are pooled from three independent experiments (mean + s.d.). *P < 0.05; **P < 0.01 (Student’s paired *t* test).

PCV2 infection can lead to immunosuppression, making the pigs susceptible to other infectious agents. Macrophages are the major target cells of PCV2 and PCV2 has been shown to replicate in mice (49, 50). We therefore determine the effect of PCV2 infection on bacterial coinfection in murine macrophages. Bacterial infection assays showed that APP or STM colony formation were significantly increased in PCV2-infected macrophages compared with that of control cells (**Figure 5A** and **Supplementary Figure 4A**, in **Supplementary Material**), indicating that PCV2 facilitates bacterial coinfection *in vitro*. To further elucidate the effect of PCV2 infection on bacterial infection, APP or STM labelled with GFP were used for bacterial coinfection assay. Fluorescence microscope analysis showed that GFP-labelled bacteria located in the cytoplasm of BMDMs in response to PCV2 infection (**Figure 5B**), suggesting that PCV2 promotes the bacterial engulfment of macrophages. Consistently, flow cytometric analysis also showed that priming of PCV2 infection increased numbers of APP-GFP or STM-GFP in macrophages (**Figures 5C, D**, **Supplementary Figure 4B** in **Supplementary Material**). Comparably, such increase was also observed when FITC-labelled latex beads was applied instead of GFP-labelled bacteria (**Figure 5E**), indicating that PCV2 infection enhances the phagocytic function of macrophages. Taken together, these data demonstrated that PCV2 infection promotes bacterial infection *in vitro*.

### Suppression of PCV2-Mediated M1 Macrophages Lowers Bacterial Infection

**Figure 6:**
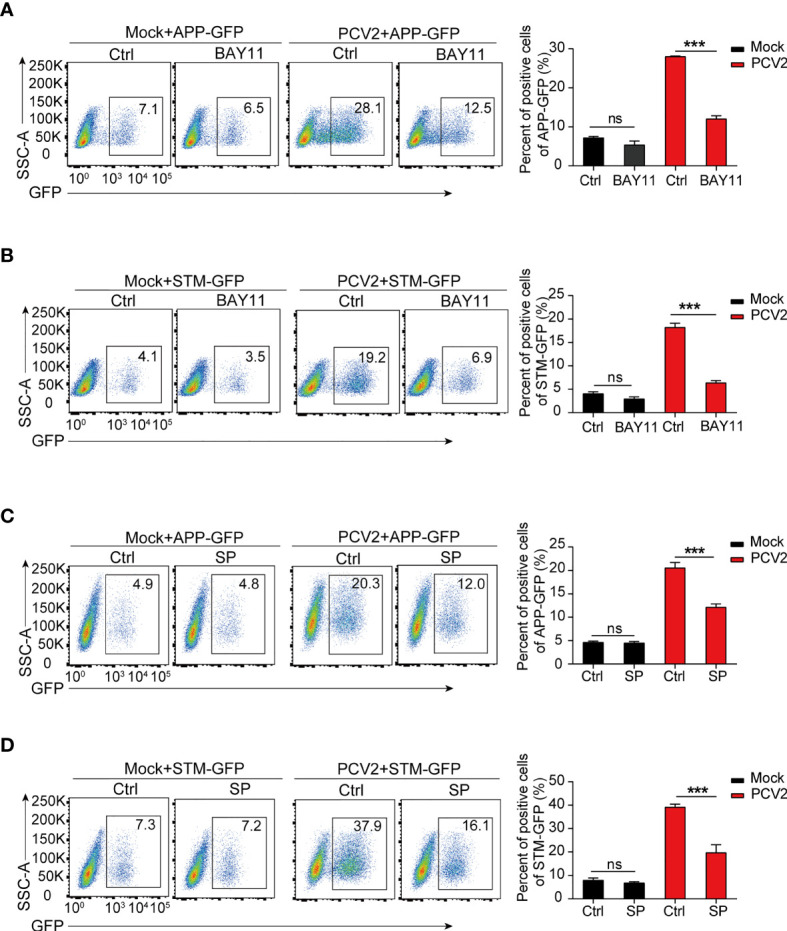
PCV2-enhanced bacterial infection depends on M1-polarized macrophages. **(A–D)** Flow cytometry analysis of GFP positive cells in BMDMs infected with APP-GFP or STM-GFP alone for 3 h or pretreated with NF-κB inhibitor (BAY11, 10 μM) or JNK inhibitor (SP: SP600126, 10 μM) for 1 h followed by APP-GFP or STM-GFP infection for another 3 h or infection with PCV2 for 12 h before APP-GFP or STM-GFP infection. ns, not significant; ***P < 0.001 (Student’s *t* test).

Having established that persistent PCV2 infection shifts macrophages in pro-inflammatory M1 status, we next sought to determine whether M1 macrophages contribute to bacterial infection *in vitro*. As mentioned above, the expression of M1-associated genes in macrophages relies on the activation of signaling cascades of NF-κB and MAPKs. Therefore, we treated macrophages with specific chemical inhibitors of NF-κB and JNK in PCV2-infected BMDMs before subsequent bacterial infection. We found that inhibition of activation of NF-κB and JNK with chemical inhibitors significantly decreased both APP or STM loads in PCV2-infected macrophages (**Figures 6A–D**), suggesting that M1 phenotype-mediated by PCV2 is critical for bacterial coinfection *in vitro*. Given that MyD88 is the key adaptor protein that leads to the activation of NF-κB and JNK signals during pathogen infection, we therefore determine the effect of PCV2 infection on bacterial infection in *MyD88*-deficient macrophages. The results showed that *MyD88* deficiency dramatically suppressed PCV2-enhanced bacterial coinfection in BMDMs compared to that in wild type cells (**Supplementary Figures 5A, B** in **Supplementary Material**), confirming the importance of PCV2-mediated M1 polarization of macrophage in promoting bacterial coinfection. As a positive control, LPS-induced M1 macrophages also promoted engulfment of bacteria *in vitro*, which is similar with the effect of PCV2 infection in macrophages (**Supplementary Figure 5C** in **Supplementary Material**).

## Discussion

In response to pathogen infection, the immune system undergoes a series of dramatic changes which may affect host defense capabilities in multiple ways (1). During PCV2 infection, the most key characteristics of PCVAD is concurrent infection with other swine pathogens. However, why PCV2 infection increases the host susceptibility to virus or bacterial coinfection remain unclear. Here, we demonstrated that PCV2 infection functionally modulated macrophage polarization to M1 state thereby promoting bacterial infection *in vitro*. Notably, PCV2 promotes expression of M1-associated genes in macrophages through activation of canonical events such as NF-κB and MAPKs that is dependent of MyD88. Moreover, PCV2 also suppresses expression of M2-associated genes *via* regulation of epigenetic histone methylation. Although recent studies indicated that PCV2-mediated expression of canonical inflammatory cytokines and inhibition of ROS production in macrophages contributed to bacterial coinfection (10, 15), the underlying mechanisms still remain obscure. Thus, our findings clarified that PCV2 infection deeply altered macrophage function to facilitate bacterial coinfection *via* a previously unappreciated mechanism by targeting macrophage polarization. Hence, the insights gained from this study advanced our understanding of the pathogenesis of PCV2. It is worthy to note that the process of PCV2 infection-induced M1 macrophage polarization not only facilitates infection of the extracellular bacteria (APP), but also promotes infection of the facultative intracellular bacteria (STM). These data suggest that the mechanisms of PCV2-mediated coinfection with bacteria are non-specific and general phenotype. Given that PCV2 is a swine pathogen, investigation the macrophage polarization mediated by PCV2 is particularly relevant for swine infection setting. In fact, our data support that PCV2 infection is also prone to maintain M1 polarization of PAMs *in vitro*. Whether the modulated function by PCV2 described here in the mouse system also are in effect in swine *in vivo* needs to be further addressed.

PCV2 preferentially targets the immune system and resides in certain antigen presenting cells including dendritic cells (DCs) and macrophages (14). Although these immune cells might not be the targets for efficient replication of the virus, persistence of PCV2 in DCs or macrophages indeed modulate the cellular immune functions, which contribute to the survival and transmission of PCV2 in swine (51). Hence, the interaction between PCV2 and immune cells may play critical roles in the pathogenesis process of PCV2. In fact, several studies have shown that, in response to PCV2 infection, porcine alveolar macrophages produce higher levels of pro-inflammatory cytokines such as TNF-α, monocyte chemotactic protein-1 (MCP-1) (52–55). Consistently, we also found that PCV2 infection in murine and porcine macrophages resulted in increased expression of a series of pro-inflammatory genes including *TNF* and *IL12B* as well as the key M1 phenotype-driven transcriptional factor *STAT1* and *HIF1α*. Of note, these genes all belongs to the feature mediators of M1-polarized macrophages. In general, M1-polarized macrophages are mainly involved in pro-inflammatory responses and functionally contribute to clearance of bacterial pathogens. Notably, the ability of M1 macrophages for clearance of bacteria is largely dependent on production of reactive oxygen species (ROS) (56). Interestingly, recent study suggested that PCV2 infection promoted APP survival during coinfection of PAMs by inhibiting ROS production *in vitro* and *in vivo (* 10). This notion was further supported by another study showing that coinfection of PCV2 and *Streptococcus suis* serotype 2 (SS2) enhances the survival of SS2 in swine tracheal epithelial cells by decreasing ROS production (20). Consistently, our results also showed that PCV2 infection induced M1 polarization and promoted uptake of bacteria. It is likely that PCV2 infection suppressed ROS production to reduce the bactericidal capacity of M1-polarized macrophages. Moreover, the replication of PCV2 is not efficient in macrophages (14). Hence, PCV2 may persistently reside in immune cells, leading to prolonged chronic inflammatory responses and tissue injury and the damaged tissues are more susceptible to secondary infections (4). In addition, PCV2 infection also suppressed expression of M2-associated transcriptional factors such as *STAT6*, *IRF4* and *KLF4*. These data support the notion that at least macrophage polarization was systemically modulated by PCV2 infection. Therefore, our finding provides direct evidence that persist infection of PCV2 shapes the macrophage immune responses to impair the local host defense and tissue damage.

Polarization of macrophages is critical in mediating an effective immune response against invading pathogens (41). Upon virus infection, M1-polarized macrophages often produce cytokines that drive virus replication and tissue damage. However, it is less clear how macrophages are reprogrammed during polarization to alter their responses. Accumulated evidence suggests that epigenetic modification of chromatin plays an important role in macrophage polarization and function (26, 45). In particular, changes of histone methylation and acetylation regulated by the corresponding epigenetic enzymes, can directly control macrophages polarization and functions (46). Consistent with this notion, we found that PCV2 infection indeed decreased the expression of histone demethylase JMJD3 in macrophages. Such regulation eventually increased the negative histone mark H3K27me3 to restrain expression of M2-assciated genes thereby sustaining M1 polarization of PCV2-infected macrophages. Thus, our data support the idea that PCV2 infection modulate M1 and M2-associated gene expression by distinct mechanisms to functionally reprogram macrophages.

Histone methylation is important for M2 polarization (46). Of note, the role of histone demethylase JMJD3 in regulation of alternative activation *via* target M2-promoting transcription factor *IRF4* by removing H3K27me3 at the *IRF4* locus has been appreciated (47). Interestingly, we found that PCV2 infection significantly reduced JMJD3 expression at both mRNA and protein levels. Mechanistically, we found that the PCV2 Cap directly binds to the promoter of *JMJD3* to suppress its transcription. Cap is the sole viral structure protein and the main antigenic determinant of PCV2, containing a nuclear localization signal (NLS) at the N terminus (57). Therefore, it is plausible that Cap regulates *JMJD3* expression *via* directly binding to the promoter in nucleus, which eventually alter the histone suppressive mark H3K27me3. Indeed, it has been known that Cap play crucial roles in viral genome packaging, capsid assembly, and virus-host interactions (15, 58, 59). Our results thus expand the regulatory function and mechanism of PCV2 Cap in viral-host cell interaction and pathogenesis. Further studies will be needed to clarify whether Cap restrains gene expression *via* regulation of other histone marks in similar ways.

In summary, our findings demonstrate that presence of PCV2 reprogramed macrophage polarization towards a M1 status *via* activation of NF-κB and JNK signaling pathways and modulation of histone methylation, which leads to enhanced bacterial coinfection (**Supplementary Figure 6** in **Supplementary Material**). These finding provides a novel mechanism of PCV2 pathogenesis and highlights the importance of persist infection of PCV2 in host immune dysfunction. Hence, modulation of macrophage polarization might be a potential therapeutic target to PCVAD in clinical.

## Data Availability Statement

The data generated in the manuscript can be accessed in GEO using the accession number GSE171721.

## Ethics Statement

The animal study was reviewed and approved by the Institutional Animal Care and Use Committees at Shandong Agricultural University.

## Author Contributions

WZ designed research, performed experiments, analyzed data, and wrote the manuscript. ZF performed bioinformatics analysis. HY helped to perform *in vivo* infection experiments. QH constructed pCMV-ORF plasmids. WF provided primary alveolar macrophages. FW provided helpful discussion. YS conceptualized the project, designed research, analyzed data, supervised experiments, and wrote the manuscript. All authors contributed to the article and approved the submitted version.

## Funding

This study was supported by National Natural Science Foundation of China (grants 32072869, 31941015), the Key Research and Development Program of Shandong Province (2019JZZY010735, 2019GNC106097) and funds from the Peak Discipline Construction Plan of Shandong Province and the High-level Talents Recruitment Program of Shandong Agricultural University (YS).

## Conflict of Interest

The authors declare that the research was conducted in the absence of any commercial or financial relationships that could be construed as a potential conflict of interest.

## Publisher’s Note

All claims expressed in this article are solely those of the authors and do not necessarily represent those of their affiliated organizations, or those of the publisher, the editors and the reviewers. Any product that may be evaluated in this article, or claim that may be made by its manufacturer, is not guaranteed or endorsed by the publisher.
