# Supplementary material for: Macrophage Polarization Modulated by Porcine Circovirus Type 2 Facilitates Bacterial Coinfection
Source: Front Immunol. 2021 Jul 28;12:688294. doi: 10.3389/fimmu.2021.688294 (PMC8355693; doi:10.3389/fimmu.2021.688294)
Supplement: Supplementary file 1 [file DataSheet_1.pdf]

# Supplementary Material

## Supplementary Figure 1

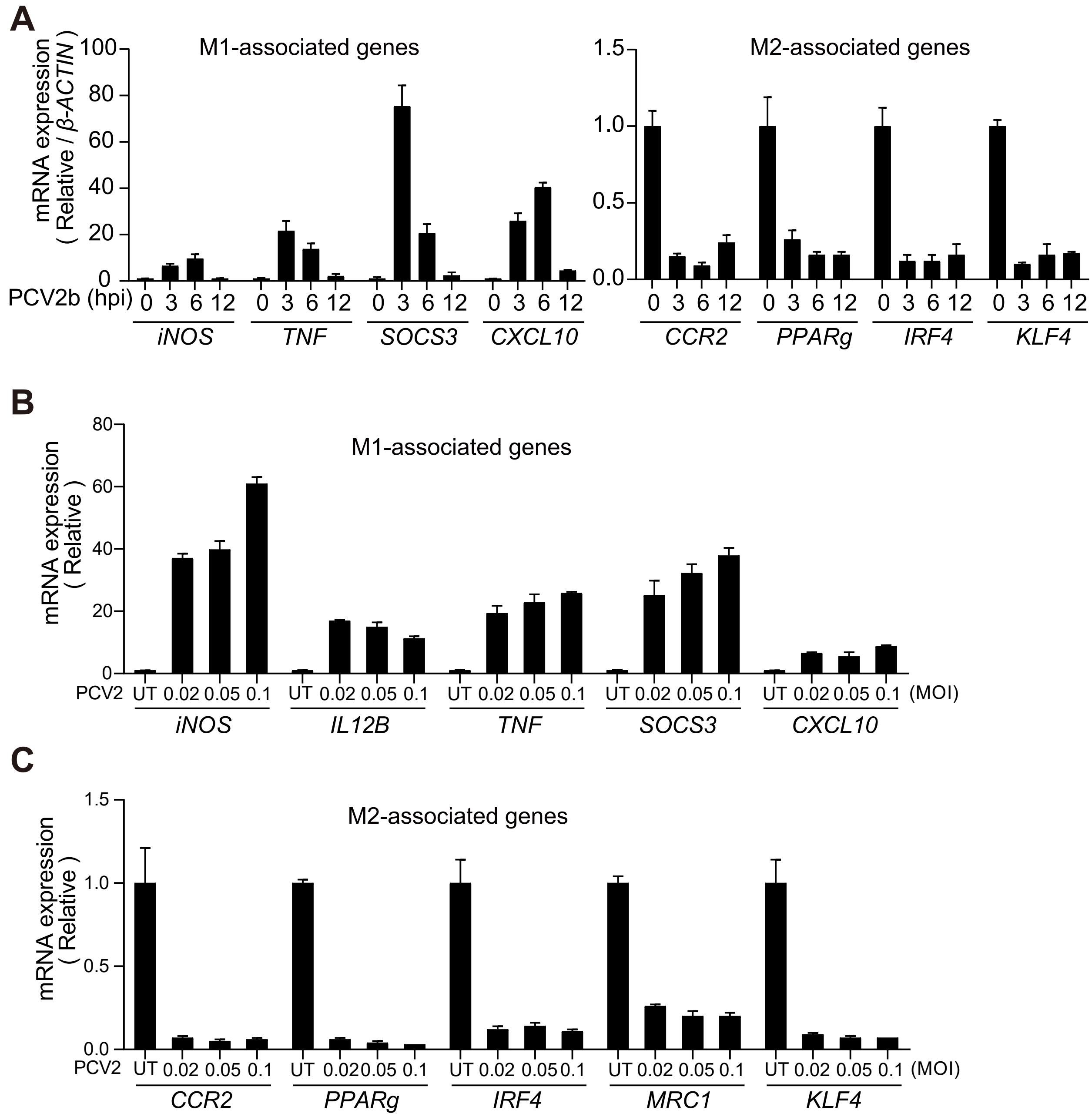


**Figure S1. PCV2 infection promotes M1 macrophage polarization *in vitro*. (A)** Quantitative real-time PCR (qPCR) analysis of mRNA expression of key M1-associated genes (left) or M2-associated genes (right) in BMDMs infected with PCV2 at indicated periods. Threshold cycle numbers were normalized to triplicate samples amplified with primers specific for the constitutive gene *β-ACTIN*. **(B, C)** qPCR analysis of mRNA expression of key M1-associated genes (B) or M2-associated genes (C) in BMDMs infected with different doses of PCV2 (MOI =0.02, 0.05 or 0.1) for 12 h, UT: untreated. Data are representative from two independent experiments (A-C, mean + s.d. of technical triplicates).

## Supplementary Figure 2

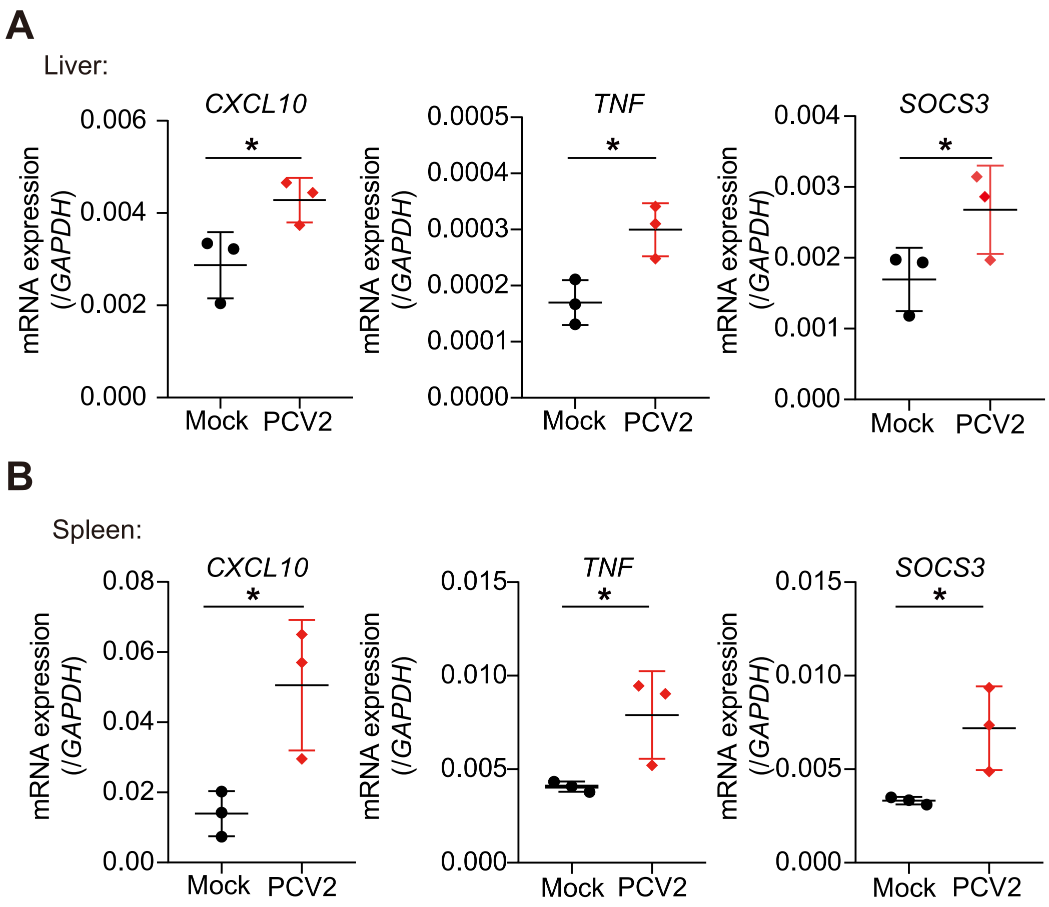


**Figure S2. Induction of M1-associated genes in PCV2 target organs. (A, B)** qPCR analysis of mRNA expression of M1-associated genes in liver (A) and spleen (B) from control mice or PCV2-infected mice ($5\times10^5$ pfu per mouse) for 24 h. Data are shown as mean + s.d. of n=3 per group. **P*<0.05 (Student's *t* test).

## Supplementary Figure 3

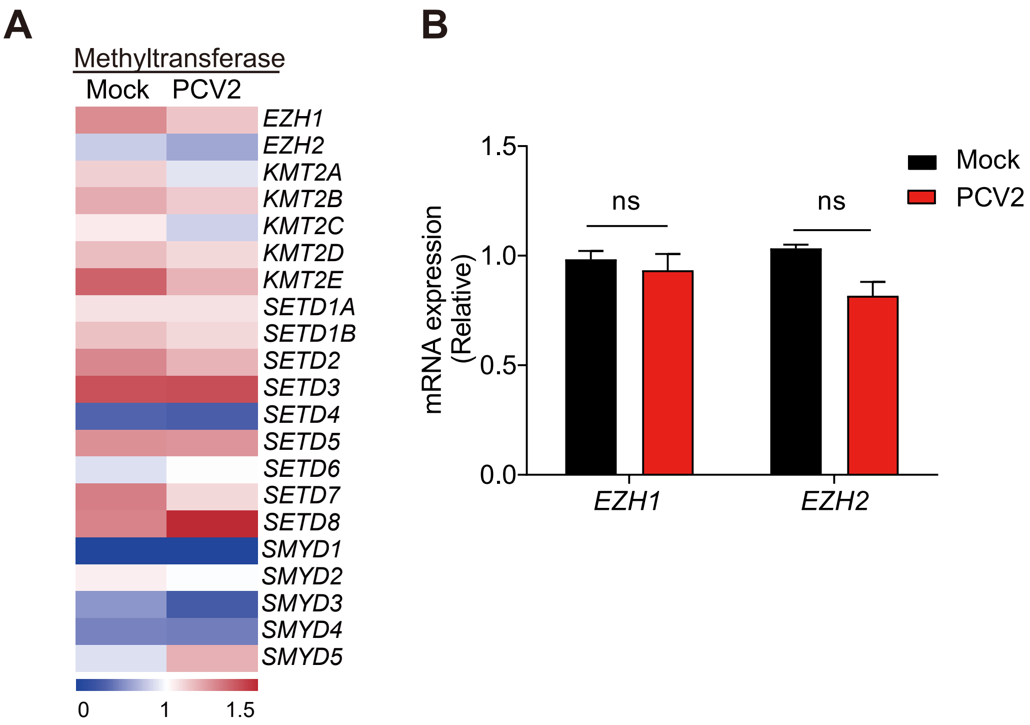


**Figure S3. PCV2 infection did not alter expression of histone methyltransferases. (A)** RNA sequencing heatmap showing genes of histone methyltransferase in BMDMs untreated or infected with PCV2 (MOI=0.02, the same dose below) for 12 h. **(B)** qPCR analysis of mRNA expression of histone methyltransferase *EZH1* and *EZH2* in BMDMs as in (A). Data are pooled from three independent experiments (B, mean + s.d.); ns: not significant.

## Supplementary Figure 4

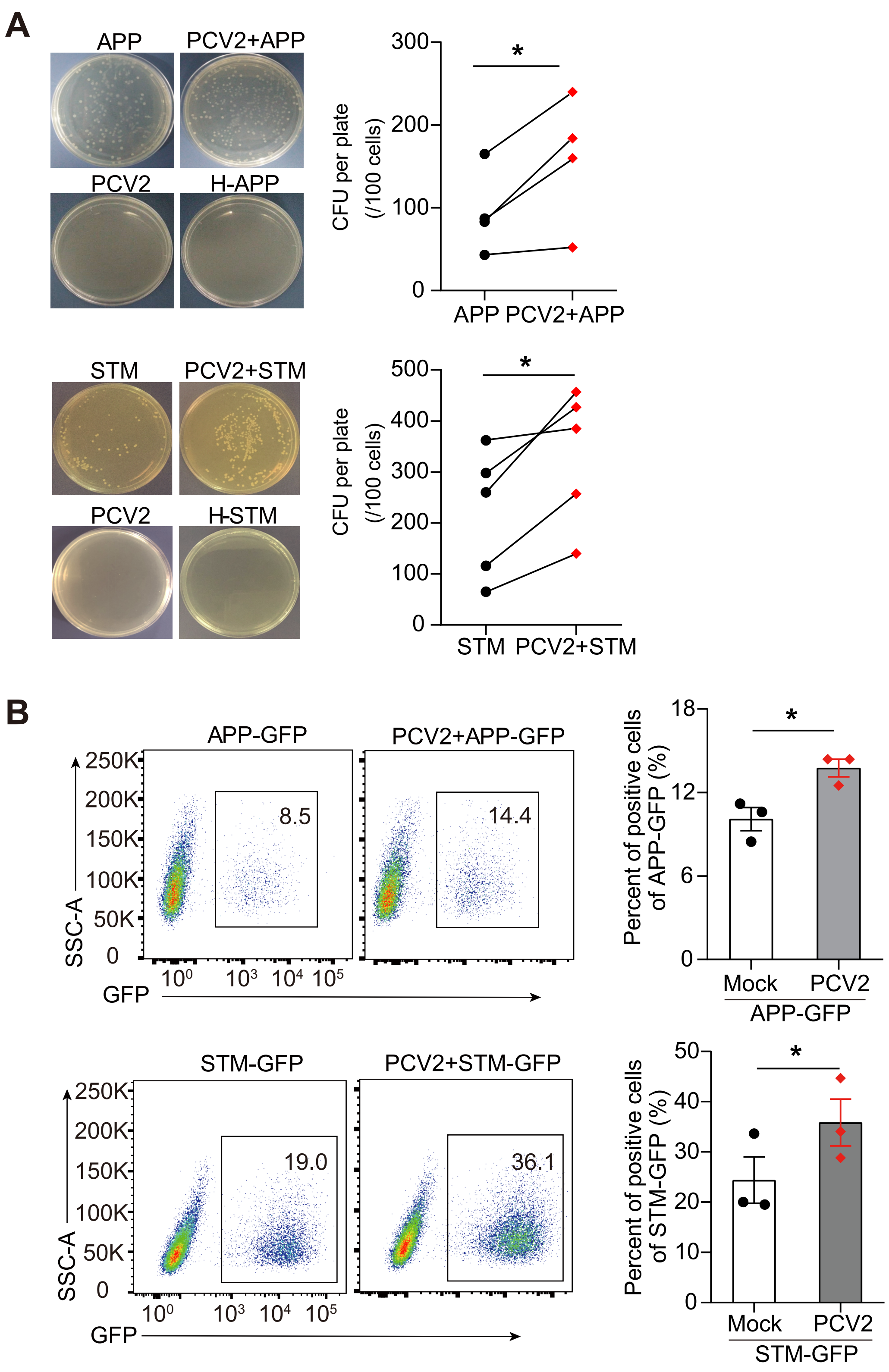


**Figure S4. PCV2 infection promotes bacterial infection in iBMDM. (A)** Colony-forming units (CFU) of APP or STM in PCV2-infected iBMDMs or control cells. iBMDMs were infected with APP or STM for 3 h. H-APP/STM: bacteria were heated at 70°C for 30 min to kill all bacteria as a negative control. Cumulative data were pooled from four or five independent experiments (right, mean + s.d.). **(B)** FACS analysis of GFP positive cells in iBMDMs infected with APP-GFP or STM-GFP alone or infected with PCV2 for 12 h followed by APP-GFP or STM-GFP for another 3 h. Cumulative data from three independent experiments is shown in right (mean + s.d.). **P*<0.05 (Student's *t* test).

## Supplementary Figure 5

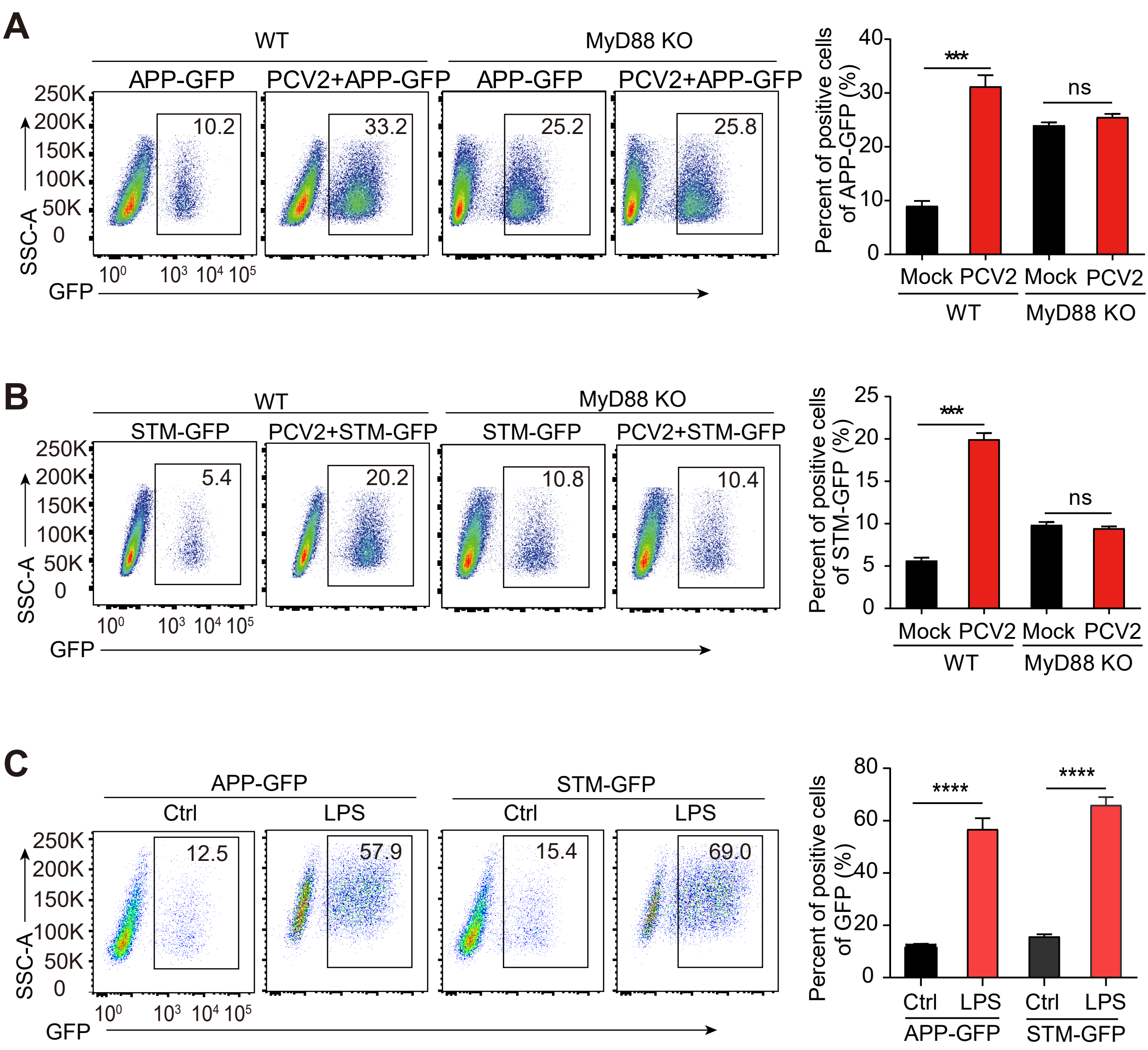


**Figure S5. Macrophage polarization is critical for bacterial infection. (A, B)** FACS analysis of GFP positive cells in BMDMs obtained from wild-type and *MyD88*-deficient mice either mock-infected or infected with PCV2 for 12 h, and subsequently inoculated with APP-GFP (A) and STM-GFP (B) for another 3 h. **(C)** FACS analysis of GFP positive cells in BMDMs stimulated without or with LPS (10 ng/ml) for 12 h followed by APP-GFP or STM-GFP (MOI=10) infection for another 3 h. ns: not significant; ***, $P < 0.001$, ****, $P < 0.0001$ (Student's *t* test).

## Supplementary Figure 6

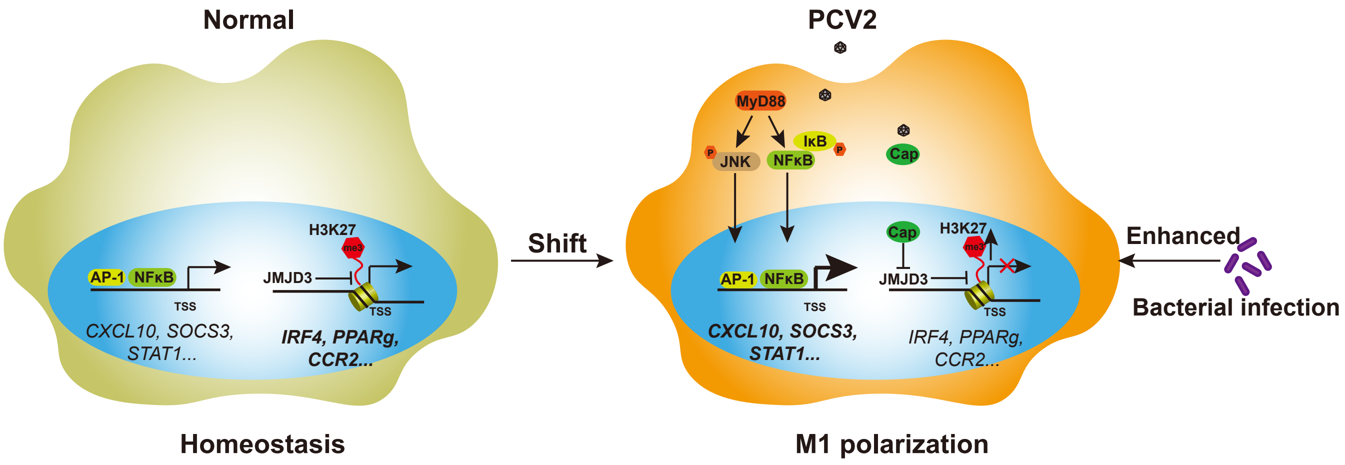


**Figure S6. A working model for M1 macrophage polarization mediated by PCV2 infection.** In resting macrophages, expression of M1 and M2-associated genes are balanced. Upon PCV2 infection, expression of M1-associated genes was induced through activation of NF-κB and JNK signals and expression of M2-associated genes was suppressed via increase of H3K27 trimethylation through inhibiting jmjd3 transcription by Cap, leading to sustained M1-polarization state that results in the enhanced susceptibility to bacterial coinfection.

| Gene | Forward Primer (5'>3') | Reverse Primer (5'>3') |
|---|---|---|
| | Primer sequences for regular qPCR | |
| *CCL5* | GCCCTCACCATCATCCTCACTG | CCTTCGAGTGACAAACACGAC |
| *iNOS* | TACTGAGACAGGGAAGTCTGAA | AGTAGTTGCTCCTCTTCCAAGGT |
| *IL12B* | AGCACTCCCCATTCCTACTTCTCC | CACCCCTCCTCTGTCTCCTTCAT |
| *TNF* | GTCAGGTTGCCTCTGTCTCA | TCAGGGAAGAGTCTGGAAAG |
| *SOCS3* | GTTTACAATTTGCCTCAATCA | TTCAAGCATCTTCAGACAGC |
| *CXCL10* | GGATCCCTCTCGCAAGGA | ATCGTGGCAATGATCTCAACA |
| *STAT1* | GCCTCTCATTGTCACCGAAGAAC | TGGCTGACGTTGGAGATCACCA |
| *IL6* | AGGCATAACGCACTAGGTTT | AGCTGGAGTCACAGAAGGAG |
| *CCR2* | GGAGAAAAGCCAACTCCTTC | AGGCAGTTGCAAAGGTACTG |
| *PPARg* | CCCTGGCAAAGCATTTGTAT | CCCTGGCAAAGCATTTGTAT |
| *IRF4* | GCCCAACAAGCTAGAAAG | TCTCTGAGGGTCTGGAAACT |
| *MRC1* | GTTCACCTGGAGTGATGGTTCTC | AGGACATGCCAGGGTCACCTTT |
| *KLF4* | GCGAGTCTGACATGGCTGT | GTCGCTTCATGTGAGAGAGTTC |
| *IL1R1* | ACCTTCCCACAGCGGCTCCACATT | TTGTCAAGAAGCAGAGGTTTACAG |
| *STAT6* | ACGACAACAGCCTCAGTGTGGA | CAGGACACCATCAAACCACTGC |
| *FIZZ1* | GGTCCCAGTGCATATGGATGAGACC | CACCTCTTCACTCGAGGGACAGTTG |
| *JMJD3* | AGACCTCACCATCAGCCACTGT | TCTTGGGTTTCACAGACTGGGC |
| *UTX* | AGCACAGAGGAGCCGTGGAAAA | GTCGTTCACCATTAGGACCTGC |
| *UTY* | CAACAGAAGTTCTGAAAGCGTGC | GGAGGATATGGCGAAGTTGGTG |
| *EZH1* | CGAGTCTTCCACGGCACCTATT | GCTCATCTGTTGGCAGCTTTAGG |
| *EZH2* | CATACGCTCTTCTGTCGACGATG | ACACTGTGGTCCACAAGGCTTG |
| *GAPDH* | ATCAAGAAGGTGGTGAAGCA | AGACAACCTGGTCCTCAGTGT |
| *β-ACTIN* | TGACAGGATGCAGAAGGAGA | GCTGGAAGGTGGACAGTGAG |
| *pCXCL10* | CCCACATGTTGAGATCATTGC | CATCCTTATCAGTAGTGCCG |
| *pTNF* | CGACTCAGTGCCGAGATCAA | CTCACAGGGCAATGATCCCA |
| *pIL12B* | TGTTCAAGTTCAGGGCAAGA | CAGGAGGAGCTGTAGTAGCG |
| *pIL6* | AAGCTGCAGTCACAGAACGA | GGACGGCATCAATCTCAGGT |
| *pSOCS3* | CACTCTCCAGCATCTCTGTC | TCGTACTGGTCCAGGAACTC |
| *pIRF4* | CCCAGCTCAGGTTCACAACT | CGGGGCACAAGCATAAAAGG |
| *pKLF4* | TGGGTGCGGAGGAACTGCTA | GCATGAGCTCTTGGTAATGGAGC |
| *pCCR2* | CTCCCTCTCTGATTCTACCT | ACAGTTCTCAAGCTCTCCAT |
| *pGAPDH* | TACACTGAGGACCAGGTTGTG | TTGACGAAGTGGTCGTTGAG |
| | Primer sequences for ChIP assays | |
| *IRF4* promoter | CCTCCGGCTCTATAAAGTTCCT | CTTACCTCACCCGCACTCTT |
| *CCR2* promoter | GTGAAACCTCTCTGCGCATTT | TTCAGCTCATGGCTTCGTCT |
| *PPARg* promoter | GATTCTCAGGCCCTCTCCAC | GGAACCGAACAAGGGTCCTC |
| *JMJD3* promoter | GGAAAGGTTGGAAGATGGCTTT | CCCCTCATTGAGAAACCAGGC |
| *HBB* TSS | CAGGGAGAAATATGCTTGTCATCA | GTGAGCAGATTGGCCCTTACC |
| | Primer sequences for plasmids construction | |
| pCMV-myc-ORF1 | CGGAATTCAGATGCCCAGCAAGAAGAATGG | CCGCTCGAGTCAGTAATTTATTTCATATGG |
| pCMV-myc-ORF2 | CGTCGACTATGACGTATCCGAGGAGGCGTT | GGGGTACCTTATTCATTAAGGGTTAAGTGAGGGG |
| pMx-myc-ORF1 | CGGGATCCATGGCATCAATGCAGAAGCTG | CCGCTCGAGTCAGTAATTTATTTCATATGG |
| pMx-myc-ORF2 | CGGGATCCATGGCATCAATGCAGAAGCTG | ATGCGGCCGCTTATTCATTAAGGGTTAAGTGAGGGG |
| pGL3-*JMJD3* promoter | GGGGTACCCCTAGCCAATCCAGTTTTCT | CCGCTCGAGAAAAGAAAACAATTTCAGTT |

**Supplementary Table 1. Primers used in this study.**
